# Boron-Doped Carbon Dots for Organelle Labeling and Mitochondrial Bioimaging

**DOI:** 10.3390/mps9030086

**Published:** 2026-06-01

**Authors:** Aasia Bibi, Daniela De Benedictis, Giuseppe Capitanio, Alessandra Gabriele, Alessandro Buccolieri, Mariapompea Cutroneo, Lorenzo Torrisi, Daniela E. Manno, Antonio Serra, Domenico De Rasmo, Anna Signorile

**Affiliations:** 1 Department of Translational Biomedicine and Neuroscience, University of Bari Aldo Moro, 70124 Bari, Italy; aasiabibi250@gmail.com (A.B.); debenedictis.d0@gmail.com (D.D.B.); giuseppe.capitanio@uniba.it (G.C.); 2 CEDAD-Centro di Fisica Applicata Datazione e Diagnostica, Dipartimento di Matematica e Fisica “E. De Giorgi”, Università del Salento, 73100 Lecce, Italy; alessandra.gabriele@unisalento.it (A.G.); alessandro.buccolieri@unisalento.it (A.B.); daniela.manno@unisalento.it (D.E.M.); antonio.serra@unisalento.it (A.S.); 3 Dipartimento MIFT-Scienze Matematiche e Informatiche, Scienze Fisiche e Scienze della Terra, Università di Messina, Viale F.S. d’Alcontres 31, 98166 Messina, Italy; mariapompea.cutroneo@unime.it (M.C.); ltorrisi@unime.it (L.T.); 4 Institute of Biomembranes, Bioenergetics and Molecular Biotechnologies (IBIOM), National Research Council of Italy (CNR), 70124 Bari, Italy

**Keywords:** nitrogen-doped carbon dots (N-CDs), boron–nitrogen co-doped carbon dots (BN-CDs), mitochondria, bioimaging, cancer

## Abstract

Background: Carbon dots (CDs) are promising fluorescent nanomaterials with great application potential in bioimaging and organelle-targeted diagnostics. This study compares nitrogen-doped (N-CDs) and boron–nitrogen co-doped CDs (BN-CDs) in normal NIH3T3 fibroblasts and KRAS-transformed cells. Methods: CDs were synthesized via a microwave-assisted method. Their fluorescence, cytocompatibility, and intracellular localization were evaluated using confocal microscopy, 3-(4,5-dimethylthiazol-2-yl)-2,5-diphenyltetrazolium bromide (MTT) assays, and organelle colocalization. Cellular metabolism was assessed by Seahorse analysis. Oxidative stress and cAMP levels were pharmacologically modulated. Results: BN-CDs exhibited stronger intracellular fluorescence than N-CDs, indicating enhanced uptake and imaging performance, with no cytotoxicity up to 100 µg/mL. They localized to multiple organelles, particularly mitochondria. However, fluorescence was significantly reduced in KRAS-transformed cells despite similar mitochondrial mass. BN-CDs did not affect mitochondrial respiration or glycolytic activity. Induced oxidative stress or elevated cAMP in normal cells reduced BN-CD fluorescence. Conclusions: Boron doping improves N-CD imaging properties without affecting cell viability or metabolism. Reduced fluorescence in KRAS cells is associated with altered intracellular conditions, suggesting that BN-CDs could be used to discriminate between normal and cancer cells.

## 1. Introduction

Carbon dots (CDs) have recently emerged as a versatile class of fluorescent nanomaterials with remarkable physicochemical properties, including high photostability, tunable emission spectra, water solubility, and low toxicity [1]. These features make them particularly attractive for biomedical applications, especially in live-cell imaging, biosensing, and targeted diagnostics [2]. Compared to traditional fluorescent probes, CDs offer improved biocompatibility and reduced environmental impact, positioning them as promising tools for next-generation bioimaging technologies [3].

A key strategy to further enhance the optical and functional properties of CDs involves heteroatom doping. In particular, boron doping has been shown to significantly modulate the electronic structure of carbon-based nanomaterials, leading to improved fluorescence quantum yield, altered surface chemistry, and enhanced interaction with biological systems [4]. Despite these advantages, the biological behavior of boron–nitrogen co-doped carbon dots (BN-CDs), especially regarding subcellular targeting and metabolic-dependent cellular responses, remains to be elucidated. Subcellular localization is a critical aspect in the development of effective nanoprobes. Among intracellular organelles, mitochondria are the central hub for cellular metabolism, energy production, and signaling pathways and are deeply involved in several pathological processes including cancer [5,6,7]. Tumor cells, in particular, exhibit profound metabolic reprogramming, including alterations in mitochondrial function, redox balance, and signaling pathways [6,8,9]. These changes may influence not only the uptake and distribution of nanoparticles but also their optical properties within the cellular environment [10,11]. In this context, understanding how nanomaterials interact with different cellular states is essential for developing reliable diagnostic tools. NIH3T3 cells transformed with the K-ras oncogene mutated at codon 12 G-C (referred to as KRAS cells) represent a well-established model of oncogenic metabolic reprogramming, characterized by altered mitochondrial activity, increased reactive oxygen species (ROS), and dysregulated signaling pathways such as cAMP-dependent processes [12,13,14]. These features provide an ideal system to investigate whether nanomaterial-based probes can discriminate between normal and cancer cells based on their metabolic phenotype. In the present study, BN-CDs were synthesized and characterized using a microwave-assisted approach. Nitrogen-doped carbon dots (N-CDs), although previously characterized and reported in preliminary studies [15], were also included to enable a direct comparison of their properties. The biological performance of BN-CDs was compared with that of N-CDs in normal NIH3T3 fibroblasts and KRAS-transformed cells. We focused on their cytocompatibility, cellular uptake, fluorescence properties, and subcellular localization, with particular attention to mitochondrial targeting. Furthermore, we investigated how specific metabolic alterations influence the fluorescence behavior of BN-CDs. Our findings provide new insights into the interaction between doped carbon nanomaterials and cellular systems, highlighting the potential of BN-CDs as multifunctional probes for subcellular imaging and as sensitive tools to detect metabolic differences between normal and cancer cells.

## 2. Materials and Methods

### 2.1. Synthesis of CDs

The synthesis was performed as follows. For N-CDs, citric acid (0.75 g) and urea (0.75 g) were used. For BN-CDs, citric acid (0.75 g), urea (0.37 g), and boric acid (0.37 g) were used. Although individually widely reported, their combined use enables a one-pot N/B co-doping strategy that leads to a chemically and electronically heterogeneous carbon network, ultimately giving rise to the observed radial electronic structure and enhanced photoluminescence. In both cases, the reagents were dissolved in distilled water to a final volume of 5 mL. For the microwave-assisted treatment, the reaction mixture was transferred into a microwave-compatible borosilicate glass tube (Chemglass Life Sciences, Vineland, NJ, USA). The tube was kept open during the microwave process, without closing the cap, in order to avoid pressure build-up and to allow safe heating under atmospheric pressure. Microwave-assisted synthesis was carried out using a Panasonic microwave oven equipped with an air-cooled magnetron (2M244-M39, 1000 W, operating at 2.45 GHz). The reaction mixtures were irradiated at 540 W (corresponding to nominal microwave energy supplied by the instrument of 324 kJ) for 10 min, leading to the formation of a brown-colored solution. After irradiation, the products were diluted with distilled water (4:1 *v*/*v*) and sonicated for 15 min to ensure complete homogenization. Sonication was performed using an ultrasonic bath sonicator (Branson Ultrasonic Cleaner 1800 Danbury, CT, USA). The samples were sonicated in water bath at a fixed frequency of 40 kHz and nominal power of 143 W for 15 min at room temperature. During sonication, the tube was kept immersed in the water bath with the bath water up to the level of the sample solution to ensure efficient acoustic coupling and homogeneous dispersion. The total energy density applied during the synthesis process was 6.48 × 10^4^ J/cm^3^. Purification was carried out through a multi-step process. First, the crude suspension was subjected to centrifugation at 3000 G (G force) to separate the supernatant from the coarse precipitate. The collected supernatant was then purified by membrane filtration using a two-step syringe filtration process using a Millex Syringe nylon filter (Merck KGaA, Darmstadt, Germany) (0.45 µm followed by 0.22 µm), effectively removing aggregates, dust, and larger particles while allowing carbon dots to pass through without retention. Finally, the filtrate underwent additional centrifugation with a relative centrifugal force of 1000 G to further eliminate any remaining impurities and obtain a homogeneous carbon dot dispersion. During microwave-assisted synthesis, the aqueous mixture of citric acid, urea, and boric acid experiences rapid and uniform heating. A temperature range of 180–220 °C is sufficient to induce dehydration, polymerization, and subsequent carbonization of the precursors. Maintaining this thermal window is crucial for the formation of carbon dots with desirable optical properties. The concentration of carbon dots in the final dispersion was determined gravimetrically by lyophilizing a known aliquot volume of the purified suspension. Specifically, an accurately measured volume (V = 1 mL) was freeze-dried under vacuum to constant weight, and the residual solid mass was used to calculate the concentration (µg/mL). This value was then used to prepare all working solutions (e.g., 50 and 100 µg/mL) by appropriate dilution with deionized water or cell culture medium. Although dialysis or SEC may provide an additional level of purification, the combination of repeated centrifugation, sequential membrane filtration, and gravimetric validation yielded stable and reproducible BN-CD dispersions suitable for the present biological investigations.

### 2.2. Characterization of CDs

UV–Vis absorption measurements of the carbon dot colloidal suspension were performed with a Cary 5000 Agilent UV–Vis–NIR spectrophotometer over the spectral range of 200–800 nm. Spectra were recorded using a quartz cuvette with an optical path length of 1 cm, while deionized water served as the reference for baseline correction. Before analysis, the carbon dots were dispersed in deionized water at a concentration of 10 mg/mL and sonicated for 10 min to ensure a uniform suspension.

Steady-state fluorescence measurements were carried out using an RF-5301 PC spectrofluorometer (Shimadzu, Kyoto, Japan). The absolute photoluminescence quantum yield (PLQY) was determined with an FLS 920 spectrometer (Edinburgh Instruments, Livingston, UK) equipped with an integrating sphere.

Transmission electron microscopy analyses were conducted using a JEOL JEM-ARM 200F NEOARM operating at an accelerating voltage of 200 kV. The carbon nanostructures were investigated in scanning transmission electron microscopy (STEM) mode using high-angle annular dark-field (HAADF) imaging. In this configuration, a finely focused electron probe was raster-scanned across the specimen, and elastically scattered electrons collected at high angles (>50 mrad) were detected with an annular dark-field detector. The resulting Z-contrast imaging enhances density variations and provides strong sensitivity to atomic number differences. Imaging conditions were optimized by setting the probe convergence semi-angle to 30 mrad and adjusting the camera length to 2 cm. Acquired images were subsequently processed with Digital Micrograph (DM) software 3.62 for noise reduction and contrast optimization.

EELS measurements were performed in scanning transmission electron microscopy (STEM) mode using a Si-STEM configuration operated at an accelerating voltage of 200 kV. Spectra were acquired with an energy dispersion of 0.05 eV/channel, enabling high-resolution detection of both low-loss and core-loss regions. The convergence and collection semi-angles were set to 5 mrad and 15 mrad, respectively, to optimize signal-to-noise ratio while preserving spatial resolution.

Low-loss and core-loss spectra were recorded in spectrum imaging mode, with dwell times of 500 ms per pixel and a step size of 0.5 nm. The zero-loss peak was used for energy calibration and deconvolution. Post-acquisition processing was carried out using Gatan Digital Micrograph. The spectra were aligned to the zero-loss peak, and plural scattering was removed using Fourier-log deconvolution. Background subtraction for core-loss edges (C K-edge and B K-edge) was performed using a power-law fit prior to edge onset.

### 2.3. Cell Cultures and Treatment with Carbon Dots

The mouse cells lines, NIH3T3 (referred to as 3T3) and NIH3T3 transformed with K-ras oncogene mutated at codon 12 G-C (referred to as KRAS), were grown in a growth medium consisting of Dulbecco’s Modified Eagle Medium (DMEM) High Glucose (Euroclone, Italy) supplemented with 10% (*v*/*v*) Fetal Bovine Serum, 4 mM Glutamine, 1% (*v*/*v*) Penicillin/Streptomycin at 37 °C and 5% CO_2_. KRAS or 3T3 cells were seeded on sterilized glass coverslips in one well of a 6-well plate (400,000 cells/well). After the cells reached 80% confluency, the growth medium was removed, and the cells were washed three times with 2 mL phosphate-buffered saline (PBS). After washing, the cells were incubated for 1 h at 37 °C, 5% CO_2_ with N-CDs or BN-CDs at 50 and 100 μg/mL in 2 mL growth medium (20 and 40 µL of 5 mg/mL CD stock solution, respectively). The CD stock solution was prepared at 5 mg/mL in autoclaved pure water. The concentrations of 50 and 100 µg/mL were selected based on preliminary screening experiments and previous literature reports indicating that this range is sufficient to evaluate the biological activity avoiding aggregation [16,17].

### 2.4. MTT Assay for Cell Viability Evaluation

Briefly, 10,000 3T3 or KRAS cells were seeded for each well in 96-well plates at 312.5 cells/mm^2^. After 24 h, the growth medium was removed, and the cells were washed three times with 100 µL PBS. After washing the cells were incubated for 1 h at 37 °C, 5% CO_2_ with N-CDs or BN-CDs at 50 and 100 μg/mL (10 and 20 µL of 5 mg/mL CD stock solution, respectively) in 1 mL growth medium. After treatment, media with CD were removed, and the cells were washed three times with 100 µL PBS. Then, 0.25 mg/mL 3-(4,5-dimethylthiazol-2-yl)-2,5-diphenyltetrazolium bromide (MTT) solution (Sigma-Aldrich) in growth medium (7.5 µL of 5 mg/mL MTT stock solution in 150 µL growth medium) was added and incubated at 37 °C for 2 h. MTT solution was removed, and crystals were solubilized in 150 µL isopropanol (Sigma-Aldrich). Absorbance was measured at 590 nm using a BioTek Cytation 5 microplate reader (Agilent, Santa Clara, CA, USA). The amount of formazan produced is directly proportional to the number of viable cells.

### 2.5. Scanning Electron Microscopy (SEM) Imaging

Morphological and elemental analyses of cells were performed using a Hitachi SU3800 scanning electron microscope (SEM) equipped with an Oxford AZTEC EDX system (Oxford Instruments, High Wycombe, UK).

Cells incubated with BN-CDs were fixed in 2.5% glutaraldehyde in PBS for 30–60 min at room temperature to preserve ultrastructure. Following fixation, samples were dehydrated through a graded ethanol series (30%, 50%, 70%, 90%, and 100%), with each step lasting 5 min, to remove water while minimizing morphological distortion. Dehydrated cells were then air-dried or critical-point-dried prior to SEM imaging. All observations were performed at low voltage (1–5 keV) to preserve cellular ultra-structure, minimize spatial charging effects, and maintain surface sensitivity, enabling observation without surface metallization or carbonization.

### 2.6. Laser Scanning Confocal Microscope (LSCM) Analysis

KRAS or 3T3 cells were seeded on sterilized glass coverslips in one well of a 6-well plate (400,000 cells/well) for fixed imaging, or on Poly-D-lysine-coated WillCo^®^ Glass Bottom Dishes (35 mm diameter, 12 mm well) for live-cell imaging. As described above, after 24 h, cells were washed three times with 2 mL PBS and incubated for 1 h at 37 °C, 5% CO_2_ with CDs (50 or 100 µg/mL) in 2 mL of growth medium (20 and 40 µL of 5 mg/mL CD stock solution, respectively). Following three further washes with 2 mL PBS, cells were incubated for 15 min at 37 °C with 0.3 µM MitoTracker Orange–Red (M7510, ThermoFisher Scientific, Waltham, MA, USA) or Golgi Tracker (CellLightTM Golgi-GFP, C10592, ThermoFisher Scientific), or LysoTracker (LysoTrackerTM Green DND-26, L7526, ThermoFisher Scientific), or Endoplasmic Reticulum (ER) Tracker [ER-TrackerTM Green (BODIPYTM FL glibenclamide), E34251, ThermoFisher Scientific]. Tracker concentrations and incubation time were as follows: Golgi Tracker, 2 µL per 10,000 cells (scaled to 60 µL for 300,000 cells), incubated overnight (around 16 h) in 2 mL of growth medium; LysoTracker, 75 nM for 30 min in 2 mL of growth medium; ER-Tracker, 1 µM for 30 min in 2 mL of growth medium. All incubations were performed at 37 °C. For LysoTracker and ER-Tracker, live-cell imaging was performed, whereas MitoTracker and Golgi Tracker-labeled cells, prior to imaging, were fixed with 4% paraformaldehyde for 10 min at 37 °C, mounted on glass slides, and imaged using a Leica confocal microscope (Leica, Wetzlar, Germany) with LAS X software 1.4.7. For confocal microscopy fluorescence image acquisition, CDs were visualized with excitation at 405 nm and emission at 528–593 nm; MitoTracker with excitation at 552 nm and emission at 600–625 nm; Golgi Tracker with excitation 488 nm and emission 508–520 nm; LysoTracker with excitation 488 nm and emission 500–524 nm; and ER-Tracker with excitation 488 nm and emission 514–520 nm. Fluorescence images were acquired using an LSCM with identical acquisition settings for all samples. Fluorescence intensity was quantified by analyzing all cells present in each field (around 5 fields per sample), and the mean fluorescence intensity per cell was calculated using Leica LAS X software 1.4.7.

### 2.7. Bioenergetic Analysis Using Seahorse XF Technology

Oxygen consumption rate (OCR) and extracellular acidification rate (ECAR) were measured as key bioenergetic parameters in 3T3 and KRAS cells using the Agilent Seahorse XFe24 analyzer (Agilent Technologies) and XF cell Mito Stress Test (Agilent) following the manufacturer’s instructions.

Briefly, after optimization of the seeding density, 800 cells/mm^2^ were seeded in XFe24 plates and incubated until complete adhesion (around 2 h) in 450 µL XF RPMI medium supplemented with 10 mM glucose, 2 mM L-glutamine, and 1 mM sodium pyruvate in a non-CO_2_ incubator at 37 °C. OCR and ECAR were measured at intervals by mixing and waiting periods using a set number of replicates per experimental point: basal OCR measurements were followed by oligomycin injection (1 µM) to inhibit ATP synthase, followed by sequential injections of carbonyl cyanide-p-trifluoromethoxyphenylhydrazone (FCCP) (1.5 μM) to uncouple mitochondrial respiration, and rotenone + antimycin A (1 µM  +  1 µM) to inhibit mitochondrial respiration. For ECAR analysis, at the end of running, 50 mM 2-deoxy-d-glucose was injected to shut down glycolysis and allow data correction for non-glycolytic medium acidification.

Mitochondrial parameters were calculated as an average of five technical replicates for each experiment as follows: basal respiration corresponds to the difference between OCR measurement and OCR plus mitochondrial respiratory chain inhibitors; coupled respiration is the difference between basal respiration and OCR measurement after oligomycin injection; maximal respiration is the difference between OCR plus FCCP and OCR plus mitochondrial respiratory chain inhibitors; and spare capacity corresponds to the difference between OCR plus FCCP and basal OCR.

ECAR parameters were calculated as follows: glycolysis is the difference between the basal measurement and the measurement after 2-deoxy-d-glucose injection; glycolytic maximal capacity corresponds to the difference between ECAR plus oligomycin and ECAR plus 2-deoxy-d-glucose injections; and glycolytic reserve capacity is calculated as the difference between ECAR plus oligomycin and ECAR basal measurement.

### 2.8. Data Analysis

The data presented in Figures are means ± SD (standard deviation). Statistical difference was determined by Student’s *t*-test. *p*-value < 0.05 was considered as statistically significant (* *p* < 0.05; ** *p* < 0.01; *** *p* < 0.001).

## 3. Results

### 3.1. BN-CD Characterization

**Figure 1:**
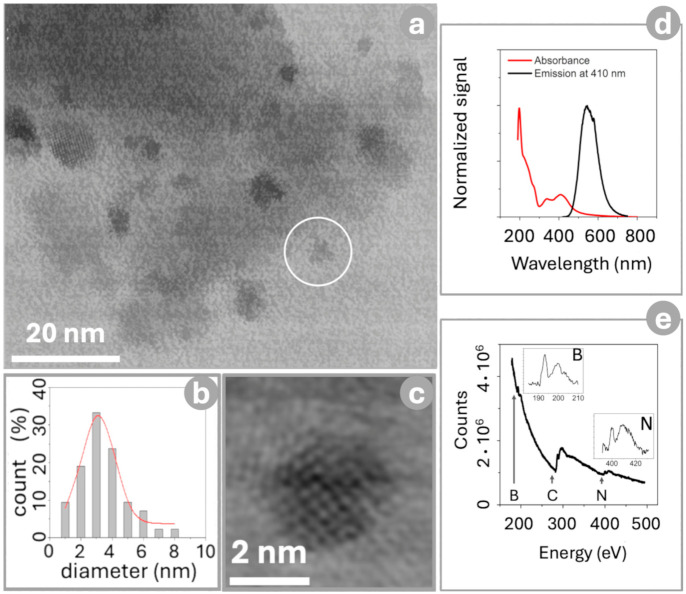
TEM and optical characterization of BN-CDs. (**a**) Low-magnification TEM image showing irregular, low-contrast carbon nanoflakes. (**b**) Histogram of nanoparticle sizes fitted with a Gaussian distribution centered at 3.25 nm with a standard deviation of 1.50 nm. (**c**) High-resolution TEM image of the nanoparticle circled in (**a**). (**d**) UV–Vis absorbance spectrum (red line) and photoluminescence emission (black line) obtained upon 410 nm excitation. (**e**) EELS spectrum showing the absorption edges of B, C, and N; the insets highlight the spectral region corresponding to the B and N edges.

Figure 1a shows a region of the BN-CD sample where carbon-based nanostructures are clearly visible. As observed, these structures exhibit low contrast with respect to the background and an irregular morphology, consistent with the presence of carbon nano-flakes.

The size analysis indicates that the nanostructures have an average size of about 3 nm, as evidenced by the size distribution reported in the histogram in Figure 1b. The distribution can be well described by a Gaussian fit, with a peak centered at 3.25 nm and a standard deviation of about 1.50 nm, indicating a relatively narrow nanoparticle size distribution.

The nanostructures also appear to be crystalline, as shown by the High-Resolution Transmission Electron Microscopy (HRTEM) image in Figure 1c, acquired on the nanoparticle circled in Figure 1a. In the high-resolution image, well-defined lattice fringes are visible, indicating the presence of local crystalline order. The spacing between the fringes is consistent with that typically observed for graphitic carbon planes, suggesting that the nanostructures are composed of nanocrystalline graphitic domains arranged in small flakes.

The extremely small size (~3 nm) suggests that these domains consist of a few graphene layers, possibly with the presence of turbostratic disorder and structurally defective edges.

Optical characterization of BN-CDs highlights the pronounced effect of heteroatom incorporation on their electronic structure. The UV–Vis spectrum (Figure 1d) shows intense deep-UV absorption (200–230 nm, π → π* transitions of sp^2^ domains) and a shoulder at 250–280 nm (n → π* transitions of C=O/C–N surface groups) [18]. B/N co-doping induces a clear red-shift, generating a new absorption band at ~410–420 nm and reducing the optical band gap to ~3.02 eV. This leads to strong yellow-green emission at 540–550 nm (Black line) with a large Stokes shift (130–140 nm), effectively suppressing self-absorption and improving optical contrast for sensing and imaging [19].

PLQY measurements under identical excitation conditions (λ_exc = 410 nm) yield 55 ± 5% for BN-CDs and 58 ± 5% for N-CDs, indicating no statistically significant change upon boron incorporation. Within this framework, the maintenance of a high PLQY (>55%) is particularly significant. the PLQY values obtained from BN-CDs used in this work are substantially higher than those generally reported in the literature for comparable green synthetic routes. Typically, N-CDs synthesized from citric acid/urea exhibit PLQY values around ~20%, while B/N co-doping leads to moderate enhancements up to ~30–40% [20,21].

Despite the narrower band gap and the formation of additional surface energy states, boron incorporation does not introduce appreciable non-radiative recombination pathways. On the contrary, the data indicate that heteroatom-induced surface passivation is highly effective in preserving radiative decay efficiency. This is further supported by the broad emission profile, which reflects a distribution of surface emissive states typical of hydrothermally synthesized carbon dots, where exciton relaxation is stabilized by surface functional groups rather than quantum confinement [22]. In this regard, ATR-FTIR analysis (Figure S1 in Supplementary Materials) confirms successful B/N incorporation and a surface rich in oxygen- and nitrogen-containing groups. Broad bands at 3000–3500 cm^−1^ and peaks at 1718–1560 cm^−1^ indicate hydroxyl/amine functionalities and C=O/C=N/C–N vibrations. Boron incorporation is evidenced by B–OH (1247 cm^−1^), B–C and B–O–C (1103–1072 cm^−1^) and B–N related bands (1419 cm^−1^ and 669–890 cm^−1^), with additional N–B–O features at 943–974 cm^−1^. Overall, the data confirm effective co-doping and explain the good aqueous dispersibility of BN-CDs.

Overall, B–N co-doping tunes the optical absorption and emission properties without compromising quantum efficiency, confirming the robustness of the synthetic strategy and the suitability of BN-CDs for optoelectronic and bioimaging applications.

Electron energy loss spectroscopy (EELS) confirms the incorporation of boron and nitrogen within the carbon dot structure through the presence of distinct B, C, and N core-loss edges (Figure 1e). The C K-edge exhibits the characteristic π* and σ* features of sp^2^ carbon, although with noticeable modifications in fine structure, including peak broadening and changes in relative intensity, indicative of altered electronic density of states and increased structural disorder. A little shift in the near-edge onset (~0.8 eV) further suggests perturbation of the electronic structure induced by heteroatom doping. The B K-edge is clearly observed, while the ELNES signatures of both B and N deviate from those of elemental or well-defined boron carbide/carbon nitride phases, indicating their incorporation in a locally disordered carbon environment associated with defective or edge sites. Collectively, these features confirm heteroatom incorporation into nanocrystalline graphitic domains and support the formation of localized electronic states that contribute to the enhanced photoluminescence of BN-doped carbon dots [23].

### 3.2. Cellular Uptake, Fluorescence Signal and Cytocompatibility of N-CDs and BN-CDs

**Figure 2:**
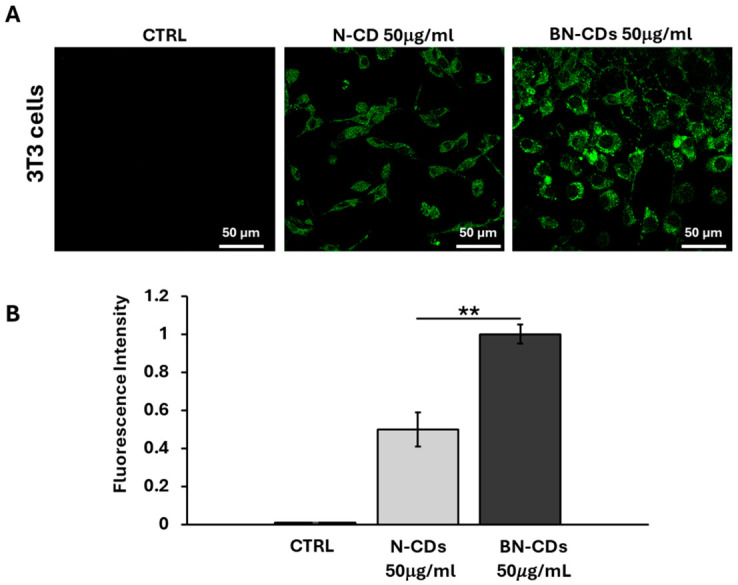
Cellular uptake and fluorescence signal of N-CDs and BN-CDs in 3T3 cells. (**A**) Representative LSCM images of 3T3 cells without CDs (control, CTRL) or incubated for 1 h with N-CDs or BN-CDs at 50 µg/mL. The images were acquired using 63x objective, excitation 405 nm and emission at 528–593 nm wavelength. The scale bars in all pictures correspond to 50 μm. (**B**) The histogram represents the quantification of fluorescence intensity, obtained by Leica confocal microscope with LASX software, in 3T3 cells treated with N-CDs or BN-CDs at 50 µg/mL. Data are presented as mean ± SD (standard deviation) of three independent experiments. Statistical analysis was performed using Student’s *t*-test (**: *p* < 0.01, BN-CDs vs. N-CDs).

In order to compare the different cellular performance of N-CDs and BN-CDs, 3T3 cells were incubated for 1 h with the two types of carbon dots at the same concentration (50 μg/mL) in the growth medium. As shown in Figure 2A, LSCM analysis showed that the BN-CDs exhibited stronger fluorescence than N-CDs, indicating improved cellular intensity of fluorescence as recorded by densitometric analysis (Figure 2B). The observed fluorescence differences at 405 nm excitation should be interpreted as imaging-performance differences obtained under identical experimental conditions rather than as a direct comparison of intrinsic emissive efficiencies for individually optimized excitation wavelengths.

**Figure 3:**
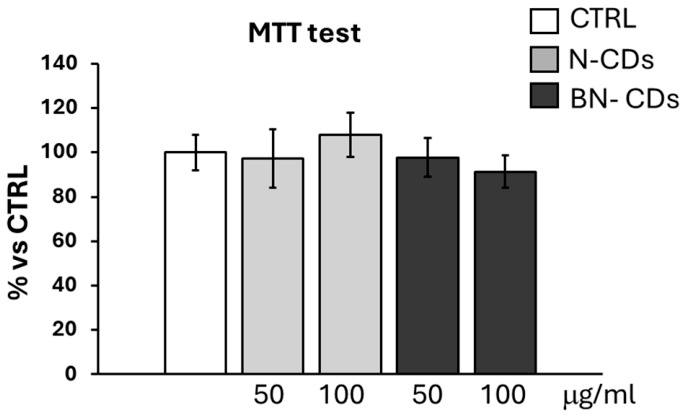
Effect of CD incubation on cell viability. 3T3 cells were incubated for 1 h in the absence of CDs (CTRL) or in the presence of 50 µg/mL and 100 µg/mL of N-CDs or BN-CDs. Absorbance was measured at 590 nm using a Cytation 5 microplate reader (BioTek). The histograms represent the means ± SD of three independent experiments. Data are presented as percentage of absorbance with respect to the CTRL. Statistical analysis was performed using Student’s *t*-test.

To test the cytocompatibility of N-CDs and BN-CDs, the MTT test was assessed in 3T3 and KRAS cell lines. The colorimetric test measures cellular metabolic activity via the reduction of tetrazolium salts in viable cells, with color intensity proportional to metabolic activity. The MTT test was performed using the two different concentrations of CDs. As shown in Figure 3, the treatment of 3T3 cells with N-CDs or BN-CDs for 1 h did not affect cell viability at both concentrations (Figure 3), which was also confirmed by LDH activity (Figure S2 in Supplementary Materials). The same tests were performed using the KRAS cell line without any effect on cell viability after N-CDs or BN-CDs treatment (Figure S2 in Supplementary Materials). The observed (not statistically significant) slight alteration in cell viability is within the expected experimental variation and does not indicate abnormal cell behavior. The results showed that under the short incubation conditions used for fluorescence imaging experiments, N-CDs and BN-CDs did not produce observable adverse effects on cell morphology or apparent cellular viability. In addition, in order to provide a more comprehensive evaluation of the cytotoxicity of N-CDs and BN-CDs, we used longer incubation times (24 h), and the results reported in Figure S3 showed no effect on cell viability. The results obtained with cytocompatibility and analysis of cellular fluorescence suggested that, using excitation at 405 nm, BN-CDs showed better biological performance for cellular image applications, and thus BN-CDs were used in the next experiments.

### 3.3. Elemental Maps of 3T3 Cells Containing BN-CDs

**Figure 4:**
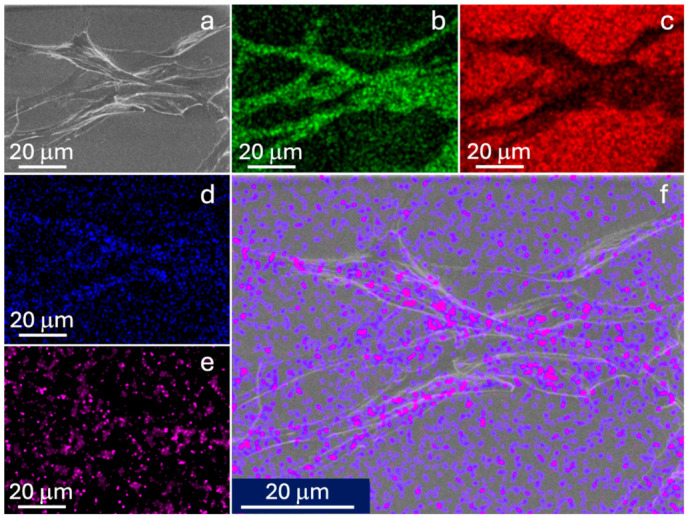
SEM analysis of 3T3 cells. Secondary electron image (**a**) showing the cell morphology, with elemental maps of carbon (**b**), oxygen (**c**), nitrogen (**d**), and boron (**e**). Panel (**f**) is a layered image highlighting the spatial localization of boron and nitrogen.

Secondary electron (SE) images were acquired to reveal the surface morphology and ultrastructural details of the cells. Complementary elemental maps of carbon, oxygen, nitrogen, and boron were obtained using energy-dispersive X-ray spectroscopy (EDS) in SEM mode. Figure 4 shows a typical example of SEM imaging analysis. Carbon and oxygen signals (Figure 4b,c) are a clear map of cells and substrate, respectively. As expected, boron and nitrogen are minor elements (Figure 4d and e, respectively). The maps reveal that, while nitrogen—which reflects both endogenous proteins and BN-CDs—follows the distribution of proteins throughout the cell and appears more delocalized, boron shows a localized accumulation in regions of approximately 2 µm within the cells, which may indicate a specific behavior or preferential uptake of the BN-CDs.

The layered image (Figure 4f) was generated by extracting and overlaying the X-ray signals for boron and nitrogen. This approach further highlights that the BN-CDs were internalized by the cells and form aggregates, suggesting a sub-localization in cellular organelles.

### 3.4. Subcellular Distribution of BN-CDs

**Figure 5:**
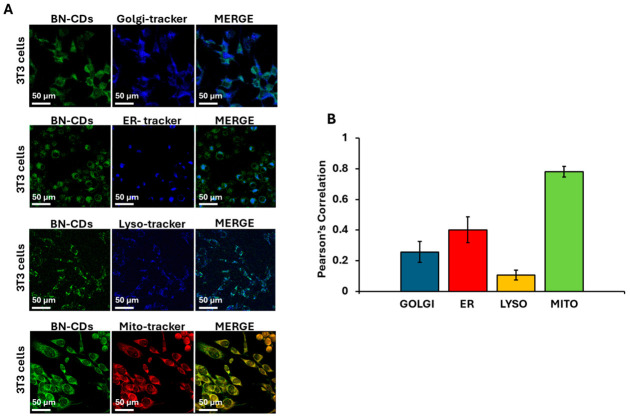
Subcellular localization of BN-CDs in 3T3 cells. (**A**) Representative LSCM images (acquired in “sequential mode” with 63x objective) of 3T3 cells co-incubated with BN-CDs (100 μg/mL; excitation: 405 nm, emission: 528–593 nm) and one of the following organelle-targeting fluorescent probes Golgi Tracker (2 µL per 10,000 cells-scaled to 60 µL for 300,000 cells; excitation: 488 nm, emission: 508–620 nm), ER-Tracker (1 μM; excitation: 488 nm, emission: 514–520 nm), LYSO tracker (75 nM; excitation: 488 nm, emission: 500–524 nm) or MITO tracker (0.3 μM; excitation: 552 nm, emission: 600–625 nm). The green fluorescence indicates BN-CDs; the blue fluorescence Golgi, ER- and LYSO Trackers; and the red fluorescence the MITO Tracker. (**B**) Data show the colocalization coefficients analyzed by Image-Pro Plus software 5.0 and expressed as Pearson correlation. The histogram represents mean ± SD of three independent experiments.

To investigate the intracellular distribution of BN-CDs, 3T3 cells were co-incubated with BN-CDs (100 μg/mL) and one of the following organelle-targeting fluorescent probes: Golgi Tracker for the Golgi apparatus, ER-Tracker for the endoplasmic reticulum, LysoTracker for lysosome and MitoTracker for mitochondrial localization (Figure 5).

The images obtained by the LSCM confirmed that BN-CDs were efficiently internalized in 3T3 cells and distributed across multiple cellular compartments (Figure 5A). Colocalization analysis showed that BN-CDs could distribute across multiple cellular compartments. Overlap between BN-CD fluorescence and MitoTracker indicated substantial mitochondrial localization. A more reduced colocalization (compared with MitoTracker) has been observed for LysoTracker, consistent with endocytic uptake pathways. Partial overlap with ER-Tracker and Golgi Tracker suggested additional intracellular trafficking routes. Quantitative colocalization analysis, using metrics such as Pearson’s correlation coefficient, further confirmed the degree of organelle-specific accumulation of CDs (Figure 5B).

### 3.5. Comparison of Cellular Fluorescence of BN-CDs in Normal and Cancer Cells

**Figure 6:**
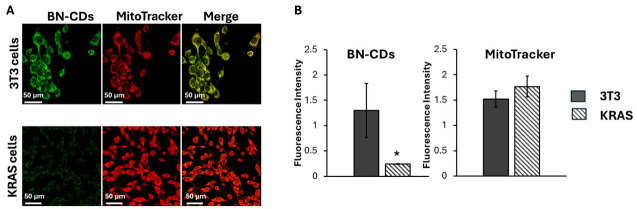
Comparison of cellular fluorescence of BN-CDs in normal (3T3) and cancer (KRAS) cells. 3T3 and KRAS cells were incubated with BN-CDs (100 μg/mL; excitation: 405 nm, emission: 528–593 nm) and co-stained with MitoTracker (0.3 µM; excitation: 552 nm, emission: 600–625 nm). (**A**) Representative LSCM images of 3T3 and KRAS acquired with 63x objective. (**B**) Quantification of intensity of fluorescence of BN-CDs and MitoTracker in 3T3 and KRAS cells. The histograms represent the mean values ± SD of the intensity of fluorescence obtained by three independent experiments. Statistical analysis was performed using Student’s *t*-test *: *p* < 0.05, KRAS vs. 3T3).

The substantial co-localization of BN-CDs with mitochondria prompted us to investigate the potential use of BN-CDs to distinguish cellular systems with different mitochondrial features. In this regard, we considered the cancer cells in which several mitochondrial deregulations have been found [7,24]. In particular, we compare 3T3 (mouse fibroblast NIH3T3 cells) and KRAS cancer cells (transformed NIH3T3-derived cell line) [25]. As shown in Figure 6A the images obtained by the LSCM showed that normal 3T3 cells stained with BN-CDs exhibited much bright green emission fluorescence with respect to cancer KRAS cells. No significant differences were observed in MitoTracker-stained cells (Figure 6B). These results suggest that BN-CDs could be used to discriminate between normal and cancer cells.

### 3.6. Bioenergetic Analysis of 3T3 and KRAS Cells Incubated with BN-CDs

**Figure 7:**
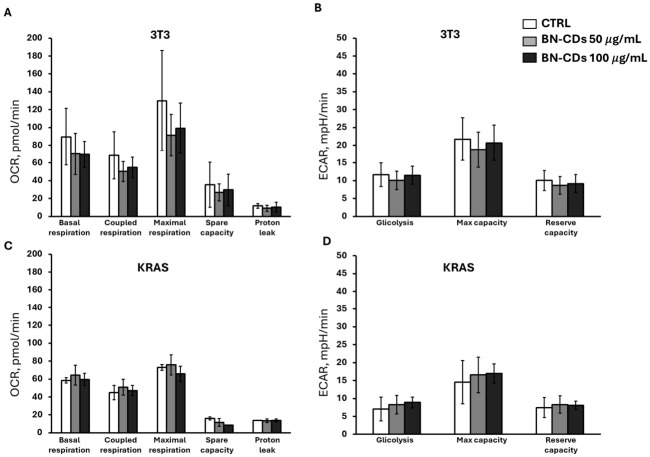
Effect of BN-CD incubation on OCR and ECAR in 3T3 and KRAS cells. 3T3 and KRAS cells were incubated for 1 h with different concentrations of BN-CDs (50 and 100 μg/mL). (**A**,**C**) OCR parameters expressed as oxygen consumption in basal condition (basal respiration), oxygen consumption linked to ATP production (coupled respiration), respiration after FCCP (maximal respiration), difference between maximal respiration and basal respiration (spare capacity) and proton leak. All parameters are expressed as pmol/min/μg protein. (**B**,**D**) Quantitative analysis of ECAR expressed as mpH/min/µg protein, derived for glycolysis, glycolytic capacity (max capacity), and glycolytic reserve (reserve capacity). See “Section 2” for experimental details. The histograms represent the mean values ± SD of three independent experiments. Statistical analysis was performed using Student’s *t*-test.

3T3 and KRAS cell lines have been previously largely characterized, showing different bioenergetic profiles in terms of mitochondrial complex I activity, mitochondrial membrane potential, ROS production, and cellular cAMP levels [12]. To investigate the effects of 1 h incubation of BN-CDs on bioenergetic profile of 3T3 and KRAS cells, a systematic analysis was carried out using Seahorse technology to assess the mitochondrial OXPHOS activity, measured as oxygen consumption rate (OCR), and the extracellular acidification rate (ECAR), primarily reflecting the conversion of pyruvate to lactate, essentially following the manufacturer’s Mito Stress protocol (Figure 7).

Neither in 3T3 nor in KRAS cells did the incubation with different concentrations of BN-CDs (50 or 100 μg/mL) for 1 h cause significant changes in OCR activity. These results were obtained by analyzing oxygen consumption under basal conditions (basal respiration), which reflect the minimal oxygen consumption required to sustain essential cellular functions; and coupled respiration, which represents oxygen utilization directly linked to ATP synthesis; and after addition of FCCP (maximal respiration) that mimics a physiological “energy demand” by stimulating the respiratory chain to operate at maximum capacity. In addition, no significant changes were found in the spare respiratory capacity, which indicates the ability of the cells to respond to stress condition and in proton leak a sign of mitochondrial damage (Figure 7A,C).

The Mito Stress Test provides information on ECAR, which predominantly reflects glycolytic flux. ECAR values, corrected for residual activity in the presence of the glycolysis inhibitor 2-DG, are reported in Figure 7B,D. BN-CD incubation of 3T3 and KRAS cells showed no significant differences in basal glycolysis, max glycolytic capacity (max capacity) or glycolytic reserve (Figure 7B,D).

Overall these results indicate that BN-CDs are efficiently taken up by cells without significant changes in bioenergetic profiles of the 3T3 and KRAS cells.

### 3.7. Cellular Alteration Affecting the Fluorescence Signal of BN-CDs

**Figure 8:**
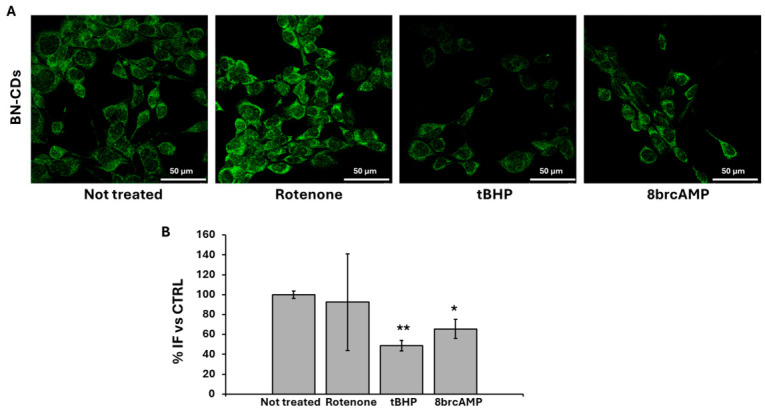
Cellular uptake of BN-CDs in different cellular altered conditions. 3T3 cells were cultured without (not treated) or with the addition of Rotenone (100 nM) for 12 h, tBHP (100 µM) for 2 h, or 8-Br-cAMP (100 µM) for 35 min. After the treatments, the cells were incubated with BN-CDs. (**A**) Representative LSCM images (63x objective, excitation: 405 nm, emission: 528–593). (**B**) Quantification of the intensity of fluorescence of BN-CDs. The histograms represent the mean values ± SD of three independent experiments. Data are expressed as percentage with respect to non-treated cells. Statistical analysis was performed using Student’s *t*-test (**: *p* < 0.01; *: *p* < 0.05, tBHP vs. non-treated, 8brcAMP vs. non-treated).

KRAS cells are cancer cells characterized by a decrease in mitochondrial complex I activity, an increase in reactive oxygen species (ROS), and increased cAMP level [12,13]. To define the cellular alterations underlying the different fluorescence signals of BN-CDs in KRAS cells with respect to 3T3, we treated 3T3 with rotenone to inhibit complex I, tert-butyl hydroperoxide (tBHP) to increase ROS, and a membrane-permeant cAMP analogue (8-Br-cAMP) to increase cAMP levels. The LSCM images and quantification analysis of fluorescence signal showed a significant decrease in cellular fluorescence in the presence of tBHP and 8-Br-cAMP compared to untreated cells, thus suggesting that oxidative stress conditions or increased cellular cAMP level could impair cellular uptake of BN-CDs (Figure 8).

## 4. Discussion

In this study, we compared the physicochemical and biological properties of N-CDs and BN-CDs, showing that boron incorporation substantially enhances their performance in cellular imaging without affecting cell viability or bioenergetic function. TEM analysis revealed that the materials consist of nanocrystalline graphitic flakes with a high density of edge defects and functionalized sites, which are expected to strongly influence both structural and optical properties. In these small graphitic domains (~3 nm), a significant fraction of atoms is located at the edges, resulting in numerous unsaturated bonds that can be passivated by heteroatoms or functional groups containing O, N, and/or B. This edge functionalization and associated structural disorder are consistent with the turbostratic disorder and defective edges observed in HRTEM, and they correlate with the observed optical behavior of the nanoparticles. Importantly, the optical characterization performed in this study was intentionally focused on the excitation region most relevant for the intended bioimaging applications. In particular, the fluorescence measurements were centered around 410 nm excitation, corresponding both to the main absorption band of BN-CDs and to the excitation wavelength employed during confocal microscopy experiments (405 nm laser line). This choice was motivated by the need to evaluate the effective imaging performance of the nanoparticles under experimentally relevant biological conditions rather than to perform a complete spectroscopic mapping of excitation-dependent emission behavior.

It is well established that carbon dots, especially heteroatom-doped systems, frequently exhibit excitation-dependent photoluminescence arising from heterogeneous surface emissive states, edge defects, and localized energy traps [22,26].

In the present BN-CDs, the incorporation of boron and nitrogen is expected to generate a distribution of surface electronic states associated with defective graphitic domains and heteroatom-related functionalities. These localized states contribute to the broad emission profile and large Stokes shift observed experimentally, which are characteristic signatures of surface-state-mediated emission mechanisms rather than purely quantum-confinement-driven photoluminescence [27,28].

Within this framework, B/N co-doping appears to induce a partial red-shift of both absorption and emission features, generating emissive states that are efficiently excitable in the violet spectral region (~405–410 nm). This aspect is particularly advantageous for confocal bioimaging applications because it enables efficient excitation using standard 405 nm laser sources commonly available in biological microscopy platforms.

Although a complete excitation-dependent fluorescence mapping could further enrich the photophysical characterization of BN-CDs, the current dataset demonstrates that the synthesized nanoparticles possess stable and intense fluorescence under the excitation conditions directly relevant to the biological imaging experiments performed in this study.

LSCM experiments revealed that BN-CDs were efficiently internalized by 3T3 cell lines, generating a significantly higher fluorescence signal than N-CDs. Cytocompatibility is an important point for the application of nanomaterials in live cell bioimaging [29,30]. Importantly, MTT assays confirmed that short-term (1 h) and long-term (24 h) exposure to both types of CDs, even at higher concentrations, does not impair metabolic activity in either normal fibroblasts or KRAS-transformed cells. These data support the cytocompatibility of the nanomaterials under the experimental conditions used and justify further studies for their application in cell imaging studies.

Subcellular localization analysis revealed that BN-CDs are distributed across multiple intracellular compartments, exhibiting partial colocalization with the endoplasmic reticulum and the Golgi apparatus, and a pronounced colocalization with mitochondria. These data suggest that, after internalization, BN-CDs undergo intracellular trafficking along the endomembrane system [31,32]. The mitochondrial accumulation of BN-CDs is most plausibly governed by a combination of electrostatic interactions, ultrasmall particle size, and surface-state effects introduced by B/N doping [33]. The primary driving force is usually the mitochondrial membrane potential. This electrochemical gradient favors the accumulation of positively charged or partially positively polarized nanostructures. If BN-CDs possess protonable amino groups (–NH_2_), amide-derived nitrogen functionalities, or positively polarized B–N/B–O surface domains (Figure S1), they may experience significant electrostatic attraction toward the negatively charged mitochondrial membrane. B/N doping can also alter the surface electronic distribution and generate localized polar regions that enhance interactions with mitochondrial membrane lipids, particularly cardiolipin, a highly anionic phospholipid abundant in the inner mitochondrial membrane [33]. This may contribute to intrinsic mitochondrial affinity even in the absence of additional classical targeting ligands. Particle size below 10 nm is another critical factor that can contribute to reaching mitochondria. The most realistic mechanism could be a synergistic combination of mitochondrial membrane potential, surface amine passivation, B/N-induced electronic polarization, and size-dependent intracellular transport [34,35]. A strong Pearson colocalization coefficient (~0.8) supports this interpretation and suggests that mitochondrial localization is not random but driven by specific physicochemical interactions.

The strong overlap with mitochondria is particularly interesting as mitochondrial targeting is highly relevant for applications in mitochondria-related diseases such as cancer. Cancer cells showed metabolic plasticity to adapt in the environment in which they develop to support proliferation [9,36,37]. It has become quite clear that a single metabolic program cannot be used to globally define altered tumor metabolism [8,38,39,40,41]. Indeed, the same type of tumor can exhibit different phenotypes with distinct responses to therapy, which may depend on various factors, including genetic, metabolic, and other molecular characteristics [40,42].

In addition, it is important to consider that cancer cells undergo significant metabolic reorganization also during disease progression, tailoring to their energy demands and fluctuating environmental conditions [43]. Furthermore, many cellular processes that involve mitochondria, such as oxidative stress, mtDNA mutation, cell apoptosis, mitochondria–nuclear communication, dynamics, autophagy, calcium overload, immunity, and drug resistance, have been found altered in cancer cells [44]. Interestingly, in the same type of tumor, different mitochondrial phenotypes can be found [8]. Considering all these aspects, the mitochondrial functionality and morphology could be considered an interesting hot spot to investigate tumor onset, progression, and response to therapy.

A key finding of this study is the differential fluorescence intensity observed between normal 3T3 cells and KRAS-transformed cells. Despite comparable mitochondrial mass, as indicated by similar MitoTracker staining, KRAS cells exhibited significantly reduced BN-CD fluorescence. This is not due to a different impact of BN-CDs on the cells; in fact, BN-CDs did not affect the viability of the cells or the metabolic profiles in either 3T3 or KRAS-transformed cells.

This suggests that the difference in fluorescence signal is not related to mitochondrial abundance but rather several parameters of cancer cells. As shown in other studies, KRAS-transformed cells showed mitochondrial complex I dysfunction, increased reactive oxygen species (ROS) production, and elevated intracellular cAMP levels [12,13].

To dissect the mechanisms underlying the reduced fluorescence in KRAS cells, we pharmacologically mimicked specific KRAS-associated alterations in 3T3 cells. The induction of oxidative stress with tert-butyl hydroperoxide and the elevation in intracellular cAMP with 8-Br-cAMP significantly reduced BN-CD fluorescence intensity, whereas complex I inhibition with rotenone had a less pronounced effect. It should be noted that, in 3T3 cells, the increase in cAMP levels slightly increases the ROS levels [12]. These results could suggest that oxidative stress may modulate fluorescence efficiency of BN-CDs. To further support this hypothesis, the fluorescence of BN-CDs was measured at an excitation wavelength of 405 in the presence or absence of H_2_O_2_ or tBHP. In agreement with the hypothesis, the results showed a decrease in fluorescence in the presence of H_2_O_2_ or tBHP, (Figure S4 in the Supplementary Materials).

However, changes in intracellular pH, redox environment, or protein corona formation, that may directly influence the photophysical properties or decrease the cellular uptake of BN-CDs in KRAS cells cannot be completely ruled out [45].

Collectively, our results demonstrate that boron doping enhances the fluorescence performance of carbon dots and reveals a differential cellular behavior between normal and cancer cells.

The present data demonstrate a strong correlation between intracellular metabolic/redox alterations and BN-CD fluorescence, while the precise physicochemical mechanisms responsible for this behavior remain to be fully elucidated. After 1 h of incubation, BN-CDs already exhibit efficient cellular internalization and clear intrinsic mitochondrial colocalization, without the need for classical targeting ligands. This indicates rapid uptake and early organelle targeting within the cellular environment. Notably, a reduced fluorescence intensity is observed in KRAS-mutated 3T3 cells compared to wild-type 3T3, suggesting an early sensitivity of the probe to oncogene-associated mitochondrial alterations, potentially related to differences in mitochondrial ROS production.

Further investigations are required to exactly elucidate the molecular mechanisms underlying BN-CD uptake, accumulation and fluorescence modulation; to assess their long-term biocompatibility; and to validate their performance across a broader range of cancer models, including in vivo systems.

## Data Availability

The data presented in this study are available on request from the corresponding authors.

## Supplementary Materials

The following supporting information (ATR–FTIR analysis; MTT test and LDH activity; MTT test after 24 h incubation; Redox interaction studies) can be downloaded at: https://www.mdpi.com/article/10.3390/mps9030086/s1.

## Abbreviations

The following abbreviations are used in this manuscript:

CDs	Carbon Dots
N-CDs	Nitrogen-Doped Carbon Dots
BN-CDs	Boron–Nitrogen Co-Doped Carbon Dots
UV-Vis	Ultraviolet–Visible
EDS	Energy Dispersive X-Ray Spectroscopy
SEM	Scanning Electron Microscopy
STEM	Scanning Transmission Electron Microscopy
TEM	Transmission Electron Microscopy
MTT	3-(4,5-Dimethylthiazol-2-Yl)-2,5-Diphenyltetrazolium Bromide
cAMP	Cyclic Adenosine Monophosphate
ER	Endoplasmic Reticulum
ROS	Reactive Oxygen Species
H2DCFDA	2′,7′-Dichlorodihydrofluorescein Diacetate
tBHP	Tertiary Butyl Hydroperoxide
8-Br-Camp	8-Bromoadenosine 3′,5′-Cyclic Monophosphate
FCCP	Carbonyl Cyanide-P-Trifluoromethoxyphenylhydrazone
OCR	Oxygen Consumption Rate
ECAR	Extracellular Acidification Rate
PLQY	Photoluminescence Quantum Yield
HAADF	High-Angle Annular Dark-Field
HRTEM	High-Resolution Transmission Electron Microscopy
PBS	Phosphate-Buffered Saline
