# Supplementary material for: Boron-Doped Carbon Dots for Organelle Labeling and Mitochondrial Bioimaging
Source: Methods Protoc. 2026 Jun 1;9(3):86. doi: 10.3390/mps9030086 (PMC13306065; doi:10.3390/mps9030086)
Supplement: Supplementary file 1 [file mps-09-00086-s001.zip › mps-4241339-supplementary.pdf]

# Boron-Doped Carbon Dots as Multifunctional Probes for Organelle Targeting and Mitochondrial Bioimaging

Aasia Bibi[1], Daniela De Benedictis[1], Giuseppe Capitanio[1], Alessandra Gabriele[2], Alessandro Buccolieri[2], Mariapompea Cutroneo[3], Lorenzo Torrisi[3], Daniela E. Manno[2], Antonio Serra[2], Domenico De Rasmo[4 †], Anna Signorile[1 †]

[1]Department of Translational Biomedicine and Neuroscience, University of Bari Aldo Moro, 70124 Bari, Italy; aasiabibi250@gmail.com, debenedictis.d0@gmail.com, giuseppe.capitanio@uniba.it, anna.signorile@uniba.it.
[2]CEDAD-Centro di Fisica Applicata Datazione e Diagnostica - Dipartimento di Matematica e Fisica "E. De Giorgi", Università del Salento, 73100 Lecce, Italy; alessandra.gabriele@unisalento.it, alessandro.buccolieri@unisalento.it, daniela.manno@unisalento.it, antonio.serra@unisalento.it
[3]Dipartimento MIFT, Università di Messina, Viale F.S. d'Alcontres 31, 98166 Messina, Italy; mariapompea.cutroneo@unime.it, ltorrisi@unime.it
[4]Institute of Biomembranes, Bioenergetics and Molecular Biotechnologies (IBIOM), National Research Council of Italy (CNR), Bari, Italy; domenico.derasmo@cnr.it

## Attenuated total reflectance – Fourier-transform infrared analysis

### Experimental

Fourier-transform infrared (FTIR) spectra were recorded using an Agilent Technologies FTIR spectrometer equipped with an attenuated total reflectance (ATR) accessory. The measurements were performed in the spectral range of 4000–600 $cm^{-1}$ with a resolution of 4 $cm^{-1}$, averaging 32 scans to improve the signal-to-noise ratio.

Before analysis, the ATR crystal (diamond) was cleaned with ethanol and dried under a nitrogen stream. A small aliquot of the BN-CDs dispersion was deposited directly onto the ATR crystal and allowed to dry to form a thin film before measurement. Background spectra were collected under the same conditions and automatically subtracted from the sample spectra.

All spectra were processed using the instrument software, including baseline correction and normalization where appropriate. The resulting FTIR spectra were used to identify the functional groups and bonding configurations present in the BN-CDs samples.

### Results

The incorporation of dopant species within both the core and surface of BN-CDs was further verified through ATR-FTIR spectroscopy. As shown in Figure S1, the FTIR spectra display a broad absorption band between 3000 and 3500 $cm^{-1}$, which can be attributed to hydroxyl and amine

functional groups present on the nanoparticle surface. Moreover, the bands located at 1718, 1679, and 1560 $cm^{-1}$ correspond to stretching vibrations of C=O/C=N and C–N bonds, suggesting the presence of carboxylic functionalities and nitrogen-containing groups.

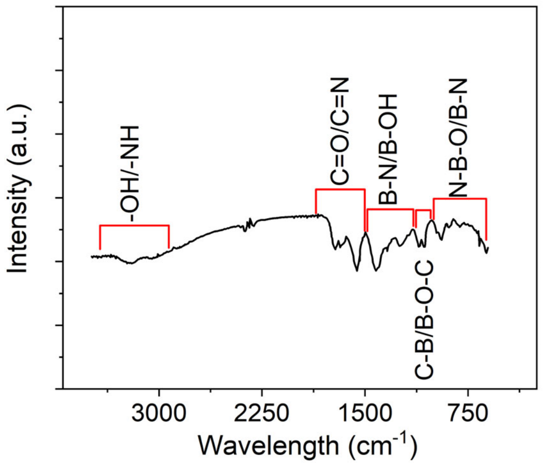


**Figure S1: Typical ATR - FTIR spectrum obtained from BN-CDs.** In combination with the B–OH stretching signal observed at 1247 $cm^{-1}$, these findings indicate that the good aqueous dispersibility of the synthesized nanoparticles is likely due to the abundance of polar surface groups. The signals at 1103 and 1072 $cm^{-1}$ are consistent with B–C and B–O–C linkages, respectively.

Additionally, the B–N stretching vibration is evidenced by a band at 1419 $cm^{-1}$, along with multiple peaks in the 669–890 $cm^{-1}$ region, while the features at 943 and 974 $cm^{-1}$ can be assigned to N–B–O bonding configurations.

## MTT test and LDH activity

### Experimental

The MTT test was performed as indicated in the Materials and Methods section. In order to evaluate the cytotoxic effect of CDs, the LDH Cytotoxicity Detection Kit of Sigma-Aldrich was used. LDH enzyme activity was determined by absorbance measurements at 490–492 nm wavelengths with an ELISA reader device.

### Results

The treatment of KRAS cells with N-CDs or BN-CDs for 1 hour did not affect cell viability at either concentration (Figure S2, Panel A). The treatments with N-CDs or BN-CDs for 1 hour did not affect LDH activities at either concentration (Figure S2, Panels B and C)

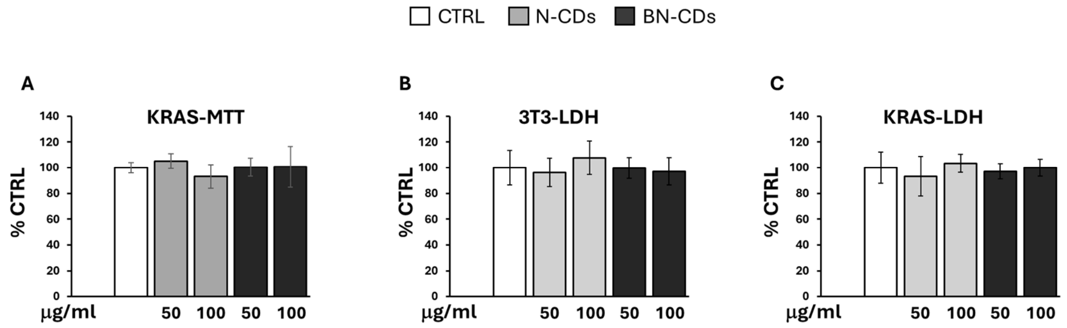


**Figure S2:** A) MTT assay results showing KRAS cell viability after 1-hour treatment with 50 and 100µg/ml N-CD or BN-CDs. B) LDH activity in 3T3 cells after 1-hour treatment with 50 and 100µg/ml N-CD or BN-CDs. C) LDH activity in KRAS cells after 1-hour treatment with 50 and 100µg/ml N-CD or BN-CDs. The histograms represent the means ± SD of three independent experiments. Data are presented as percentage of absorbance with respect to the CTRL. Statistical analysis was performed using Student's *t*-test.

## MTT test after 24 hours of incubation.

### Experimental

The MTT test was performed as indicated in the Materials and Methods section after 24 hours of incubation.

### Results

The treatment of 3T3 (panel A) and KRAS (panel B) cells with N-CDs or BN-CDs for 24 hours did not affect cell viability at either concentration (Figure S3).

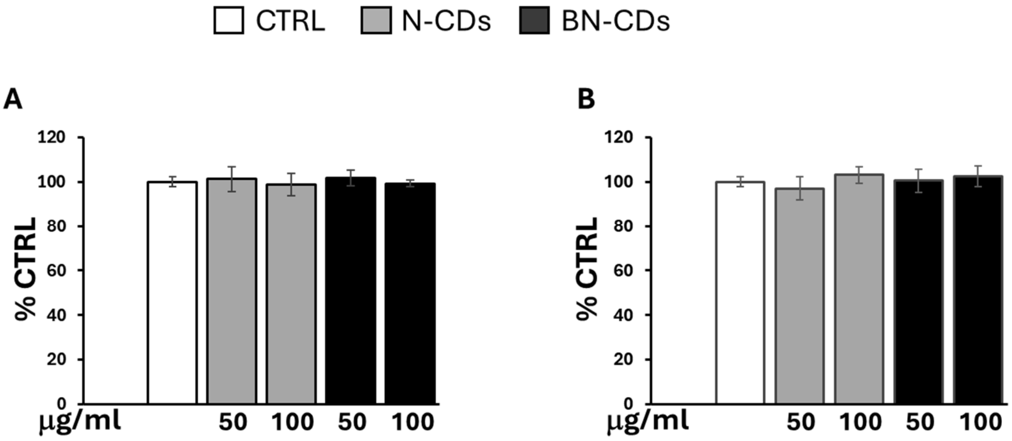


**Figure S3**: A) MTT assay results showing 3T3 and KRAS cell viability after 24-hour treatment with 50 and 100 μg/ml N-CD or BN-CDs.

## Redox interaction studies

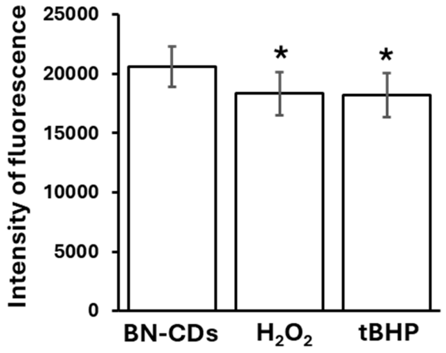


**Figure S4**: 1 µl BN-CDs (stock solution 5 mg/ml) was added to 200 µl of DMEM in 96-well plates, in the absence of cells, without (BN-CDs) or with the addition of 100 µM hydrogen peroxide ($H_2O_2$) (1 µl of a 20 mM stock solution) or 100 µM tert-butyl hydroperoxide (tBHP) (1 µl of a 20 mM stock solution). After 1 hour of incubation, the intensity of fluorescence was measured by Cytation 5 (excitation 405, emission 550 ± 20). The histograms represent the means ± SD of intensity of fluorescence of four independent experiments. Statistical analysis was performed using Student's *t*-test (*: $p < 0.05$, $H_2O_2$ *vs* BN-CDs, tBHP *vs* BN-CDs).
